# Adjuvant Therapy: Melanoma

**DOI:** 10.1155/2011/274382

**Published:** 2011-12-19

**Authors:** Diwakar Davar, Ahmad Tarhini, John M. Kirkwood

**Affiliations:** ^1^Division of General Internal Medicine, University of Pittsburgh Medical Center, 200 Lothrop Street, Pittsburgh, PA 15213, USA; ^2^Division of Hematology-Oncology, University of Pittsburgh Medical Center, 5150 Centre Avenue, Pittsburgh, PA 15232, USA

## Abstract

With an incidence that is increasing at 2–5% per year, cutaneous melanoma is an international scourge that disproportionately targets young individuals. Despite much research, the treatment of advanced disease is still quite challenging. Immunotherapy with high-dose interferon-**α**2b or interleukin-2 benefits a select group of patients in the adjuvant and metastatic settings, respectively, with significant attendant toxicity. Advances in the biology of malignant melanoma and the role of immunomodulatory therapy have produced advances that have stunned the field. In this paper, we review the data for the use of interferon-**α**2b in various dosing ranges, vaccine therapy, and the role of radiotherapy in the adjuvant setting for malignant melanoma. Recent trials in the metastatic setting using anticytoxic T-lymphocyte antigen-4 (anti-CTLA-4) monoclonal antibody therapy and BRAF inhibitor therapy have demonstrated clear benefit with prolongation of survival. Trials investigating combinations of these novel agents with existing immunomodulators are at present underway.

## 1. Introduction

The therapeutic armamentarium for melanoma has expanded recently to include several promising agents. However, there remains a significant fraction of patients with advanced disease for whom treatment options are unsuccessful. The incidence of melanoma has increased steadily over the years (currently representing the fifth most common cancer in men and the seventh most common cancer in women), increasing at a rate greater than any other human cancer. Melanoma remains a deadly disease that disproportionately targets young individuals in their prime, taking a societal toll that is greater than many other more common malignancies, such as prostate carcinoma.

For patients with surgically resected melanoma of a primary tumor thickness of 4 mm or greater (T4 lesions) and/or regional lymph node metastases who are at increased risk of recurrence and death (stages IIB or greater), the only Federal Drug Administration (FDA) approved effective adjuvant therapy remains interferon-*α* (IFN-*α*).

The standard therapy for patients with metastatic disease has been dacarbazine. Its oral analogue temozolomide has shown equivalent benefit, but has not been approved by the FDA for treatment of melanoma. [1, 2] Response rates with dacarbazine have consistently been less than 10% in recent randomized controlled trials and are generally transient. This hitherto stagnant field has seen the advent of two promising new agents that offer much hope to patients and physicians alike. Ipilimumab (MDX-010, Yervoy), a fully human monoclonal antibody (IgG1) that blocks the T-cell surface protein CTLA-4 that has immunoregulatory functions, has demonstrated a survival advantage for ipilimumab given at 3 mg/kg every 3 weeks × 4 against a vaccine comparator for second-line therapy that led to fast-track approval from the FDA for treatment of metastatic melanoma [3]. The recent presentation of data from the phase III first-line trial of ipilimumab given with dacarbazine compared against dacarbazine alone has confirmed the benefit of ipilimumab with improved response, progression free and overall survival, and with an increment of 10% in the fraction of patients surviving at 2-3 years that is similar to the results of the second-line trial MDX 10–20 that was published in 2010. These data have led to FDA approval of ipilimumab in March of 2011 [4]. The BRIM 3 study published in June 2011 [5] has demonstrated a progression free and overall survival advantage for treatment with vemurafenib (Zelboraf) compared to dacarbazine—this inhibitor of the oncogenic BRAF kinase received FDA approval in August 2011. Other treatment options in the metastatic disease setting include high-dose interleukin 2 (IL-2), which achieves durable long-term complete responses in a small proportion of patients treated but has yet to be formally compared to dacarbazine in a randomized phase III study. 

Various combinations of biological agents and chemotherapy (“biochemotherapy”) have been tested in phases II and III settings. Several have demonstrated increases in objective response rates at initial single institution phase II evaluation, but none have demonstrated survival benefit in randomized phase III trials [6]. 

This review paper will focus on recently published advances on the adjuvant treatment of high-risk melanoma. It will update the data since prior reviews published in 2010 [7, 8] and include discussion of several recent meta-analyses [9–12].

## 2. Materials and Methods

### 2.1. Search Strategy and Selection Criteria

A systematic search strategy was utilized to interrogate the Medline, Embase, Cancerlit, Cochrane, ISI, and Web of Science databases for articles published between January 1, 2002 and February 1, 2011. MeSH headings used included “melanoma, advanced,” “melanoma, adjuvant,” or “melanoma, interferon” for trials conducted in the adjuvant setting. Searches were limited to clinical trials and publications in English or with available English translations. The “related articles” feature of PubMed was used for all reports that met the requested criteria as an additional means of identifying potentially relevant investigations. Data from recently published and ongoing phase I/II/III trials were gathered by searching clinical trial databases. The abstract databases of the American Society of Clinical Oncology (ASCO) and European Society for Medical Oncology annual congresses were also searched for recently released clinical trial data. Additionally, the references in reviewed articles were analyzed to find further relevant publications.

## 3. Discussion

### 3.1. Indications for Adjuvant Therapy

Adjuvant therapy has traditionally been reserved for those postoperative patients at high risk of developing advanced disease. Research has attempted to define the clinical and pathologic features that predict risk of relapse, metastasis, and overall survival (OS). Currently, adjuvant therapy with high-dose IFN alfa-2b is the standard of care for patients with resected node-positive melanoma (stage III) and should be considered for patients with node-negative disease with a high risk of recurrence, that is, deep primary tumors (T3b, T4 a/b) whose estimated risk of recurrence exceeds 30% [13].

### 3.2. Clinical Predictive Factors

Five factors have demonstrated independent predictive value in relation to relapse and mortality based on observations of patients in the 2008 American Joint Committee on Cancer (AJCC) Melanoma Staging Database. These findings were incorporated into the revised 2009 classification on the staging and prognosis of cutaneous melanoma copublished by the AJCC and the International Union against Cancer (UICC).

The single most important factor for localized melanoma is the *depth of the primary tumor* (Breslow's tumor thickness). As tumor thickness increases, the 5- and 10-year survival rates decline. While 10-year survival is 92% among patients with T1 primary lesions and 80% among patients with T2 lesions, it drops to 63% in patients with T3 melanomas (2.01 to 4.00 mm thick) and further falls to 50% in patients with T4 tumors that are more than 4.00 mm thick. 

The *presence of primary tumor ulceration* (defined as the absence of intact epidermis overlying a significant portion of melanoma in microscopic analysis) is well known to adversely affect survival. Survival rates of patients with an ulcerated melanoma are proportionately lower than those of patients with a nonulcerated melanoma of equivalent T category but are very similar to patients with a nonulcerated melanoma of the next highest T category. The presence of primary tumor ulceration upstages each T category and is designated by the addition of “b” in conjunction with the T1-4 classification. As an example, T2b, ulcerated melanomas (1-2 mm in thickness) have a 5-year survival of 82%, while the survival for the deeper T3a category without ulceration is 79%—and both are grouped in the stage IIA category accordingly. 

Analysis of the AJCC Melanoma Staging Database data demonstrated that the *mitotic rate* was the second most powerful predictor of survival for localized melanoma after tumor thickness. Increasing mitotic rate (at least one mitosis per square millimeter) is strongly correlated with diminished survival rates and is now a component of the 7th edition melanoma staging system. It has also replaced the Clark level of invasion as a primary criterion for defining T1b melanoma—which is now defined as those lesions whose tumor thickness is ≤1.0 mm containing at least one mitosis per square millimeter regardless of tumor ulceration.

*Regional metastases* clinically evident as lymphadenopathy or intralymphatic (satellite or in-transit) metastasis are important predictors of outcome. The 7th edition AJCC staging system abolished the concept of a minimum threshold of lymphatic tumor burden defining the presence of regional nodal metastases. Specifically, lymph node tumors of less than 0.2 mm that were previously ignored in staging nodal disease were felt to be biologically and clinically significant and were now included in definition of nodal disease. This measure, coupled with the criterion that nodal micrometastases could be defined by immunohistochemical staining rather than by H&E alone, underscored the importance of microscopic involvement of lymph nodes rather than the size of nodal involvement in predicting survival. This is best illustrated by the 5-year survival of stage III patients, which subdivided according to extent of lymph node involvement show a steady decline from 78% to 59%, and 40% for stages IIIA, IIIB, and IIIC, respectively. 

For systemic metastatic disease, the *number* of metastatic sites, the *sites* of distant metastases, and the *serum lactate dehydrogenase (LDH) enzyme level* are important prognostic factors. Elevated LDH levels are known to herald a more malignant phenotype of the disease, and its importance in this regard is reflected in the M1c designation (includes nonlung visceral metastases) of the 7th edition AJCC staging system. One-year survival of patients with M1c disease is 33%, as compared to 62% for M1a melanomas (distant skin, subcutaneous, and lymph node metastases) and 53% for M1b melanomas (lung metastases). Recent work using immunohistochemical analysis of LDH expression in tissues of nevi and melanomas has shown that LDH expression is closely related to the progression of melanomas—being barely detectable in nevi but strongly expressed in thick primary melanoma and in metastatic melanoma [14].

### 3.3. IFN Therapy

An English virologist (Isaacs) and a Swiss researcher (Lindenmann) discovered IFN after noticing that heat-inactivated influenza virus inhibited the growth of live influenza virus *in vitro* in 1957.

In the next two decades, multiple experiments suggested that interferons had antitumor effects in a broad range of laboratory models. Following the purification of interferons and the subsequent cloning of interferon genes in the 1980s, it became clear that far from being a single molecular species, interferons comprise a large family of structurally related molecules with diverse biological effects. Once the interferon gene was inserted into bacteria using recombinant DNA technology [15], it was a mere matter of time before the commercial applications of interferon were discovered. 

IFNs are subclassified as types I and II according to their structural and functional properties. Type II IFNs (IFN-*γ* in humans) are released by Th1 cells. Signaling via the IFN-*γ* receptor (IFN-*γ*R), IFN-*γ* recruits leukocytes to infected areas resulting in inflammation, stimulates macrophages to phagocytose engulfed bacteria, and upregulates the Th2 response. Type I IFNs comprise a number of structurally similar molecules that all signal via the IFN-*α* receptor (IFN-*α*R). Whilst several subtypes have been identified, IFN-*α*, IFN-*β*, and IFN-*ω* are the most important ones in humans. Type I IFNs are produced in large quantities chiefly by the plasmacytoid dendritic cell in response to infectious and other noxious stimuli. Connecting the adaptive and innate arms of the immune response, type 1 IFNs have potent immunoregulatory, antiproliferative, differentiation-inducing, apoptotic, and antiangiogenic properties.

#### 3.3.1. IFN Therapy: Mechanism

The mechanism(s) by which IFNs exert antitumor effects in melanoma is not fully known. Evidence from both animal and human studies suggests that the effects of IFN-*α* are secondary to immunomodulatory effects rather than direct cytotoxic mechanisms. In a trial that tested neoadjuvant high-dose interferon (HDI) given prior to definitive lymph node dissection for patients with stage IIIB/C disease, investigators demonstrated that HDI resulted in a brisk influx of T lymphocytes and dendritic cells into the tumor in a fashion that directly correlated with response rates [16]. 

Subsequent analysis showed that HDI downregulates the MEK/ERK MAPK pathway that plays a role in tumor cell metastasis [17]. In addition, interferon also appears to downregulate STAT3—a critical progression marker in cancer cell survival, proliferation, angiogenesis, metastasis, and immune evasion [18, 19].

#### 3.3.2. IFN Therapy: Clinical Trials to Assess Dose

Early trials investigating the use of immunologically active compounds in a variety of human malignancies bore mixed results but provided the scientific rationale for further investigating the role of immunological mediators in the treatment of human malignancies. Several reports in the early 1980s suggested that IFN-*α* therapy resulted in objective responses in melanoma—galvanizing the oncological and pharmaceutical community to assure commercial production of the agent, using rDNA technology. Currently, three subspecies of IFN-*α* are available commercially: IFN-*α*2a (Roferon-A, Roche Pharmaceuticals, Nutley, NJ), IFN-*α*2b (Intron A, Schering Plough, Kenilworth, NJ), and IFN-*α*2c (Berofor, Boehringer Ingelheim, Vienna, Austria).

A multitude of phase II trials followed—testing various doses schedules and routes of recombinant and nonrecombinant IFN-*α* in metastatic melanoma to attempt to identify an optimal dose, schedule, and treatment duration with acceptable toxicity to induce response in metastatic melanoma (see Table 1). Response rates with IFN (approximately 16%) were similar to those seen with single-agent chemotherapy, but already durable responses were seen in some subjects, lasting years. It was noted that response rates were higher in patients with smaller disease burden, suggesting that the most effective results might be obtained in patients with microscopic disease treated in the adjuvant setting.

A flurry of trials examined the role of adjuvant IFN therapy for high-risk melanoma. These trials are summarized below (see Table 2) divided based on IFN-*α*2 dose: low-dose (<3 MU/dose), intermediate dose (5–10 MU/dose), and high dose (>10 MU/dose).

#### 3.3.3. IFN Therapy: Clinical Trials of High-Dose IFN

Two high-dose regimens suggested promise in North American trials completed in 1990. The North Central Cancer Treatment Group (NCCTG) trial tested a high 20 MU/m^2^ dose of IFN-*α*2a administered intramuscularly thrice weekly for twelve weeks for stage II and III disease [20]. Median disease free survival (DFS) and overall survival (OS) were improved with treatment but did not achieve statistical significance; the lower stage II patients did not appear to benefit as much as the higher risk patients with this adjuvant therapy. The second trial—the E1684 Eastern Cooperative Group (ECOG) trial—tested an induction phase of one month of daily intravenous (IV) IFN-*α*2b, followed by a prolonged (11 months) maintenance therapy with doses that approached the maximum tolerable dosage given subcutaneously (SC) [21]. 

E1684 was the first randomized controlled trial to show a significant prolongation in both DFS and OS among patients with deep primary tumors (>4 mm, T4N0M0), or the presence of regional lymph node metastases (TxN1-3M0, AJCC stage III). Notably, the trial required that all patients undergo pathologic staging of regional lymph nodes before enrolment and excluded in transit, satellite, or extracapsular spread of disease. Patients received an induction phase consisting of IFN-*α*2b IV at 20 MU/m^2^ daily for 5 days per week for 4 weeks followed by a maintenance phase of thrice weekly SC injections at 10 MU/m^2^ for 48 weeks (HDI) versus close clinical follow-up. After a median follow-up period of more than 6.9 years, there were significant differences in relapse and survival; the estimated 5-year relapse free survival (RFS) in the treatment arm was 37% (95% confidence interval [CI], 30–46%) versus 26% (95% CI, 19–34%) in the control group, while the 5-year OS in was 46% (95% CI, 39–55%) versus 37% (95% CI, 30–46%) in the treatment and observation arms, respectively.

Subgroup analysis found that patients with deep primary, node-negative melanoma (T4N0Mx) were underrepresented (11% of the total number, 280). Analysis also revealed that the node-positive patients (stage III disease) benefited the most from IFN-*α*2b therapy, with the greatest reduction of relapse early during the first several months of treatment. In fact, the greatest improvement in survival (hazard ratio) was seen in patients with clinically node-negative but pathologically positive nodes (N1 disease). The results of this groundbreaking trial led the United States Food and Drug Administration (FDA) to approve IFN-*α*2b for commercial use, and IFN-*α*2b under the ECOG 1684 protocol became the standard of care for high-risk operable melanoma patients.

HDI with a 4-week induction phase followed by a 48-week subcutaneous maintenance phase remains the only adjuvant therapy to date that *has demonstrated survival benefit in addition to durable relapse-free survival rates in two independent randomized cooperative group studies*. Significant treatment-related adverse events (AEs) raised the question as to whether low-dose IFN-*α* given for a longer duration would be similarly effective with an improved toxicity profile. 

ECOG and the US intergroup therefore compared therapy with HDI for 1 year versus low-dose IFN-*α*2b (LDI) (thrice weekly SC injections at 10 MU/m^2^) for 2 years versus observation in E1690 [22]. RFS was significantly improved in the HDI population versus observation (HR = 1.28, *P* = 0.025). Although LDI was associated with a reduced fraction of grade 3/4 AEs compared to HDI (1 [0.5%] versus 17 [8.0%] grade 4 AEs, resp.), LDI failed to achieve statistically significant durable improvement in RFS. Neither LDI nor HDI appeared to have any durable impact on OS in this trial. Retrospective analysis revealed that 37 patients had crossed over from the observation arm to the HDI arm off-protocol at the time of regional recurrence—which may have attenuated any apparent survival benefit. 

E1694 was an intergroup US study that accrued 880 patients and was designed to evaluate the benefit of vaccination with the ganglioside GM2/keyhole limpet hemocyanin vaccine (GMK) in relation to HDI [23]. The GMK vaccine consisted of purified ganglioside GM2 coupled to keyhole limpet hemocyanin (KLH). Vaccination induced antibodies against GM2 that were capable of specifically binding GM2 and killing melanoma cells *in vitro* through complement or antibody-dependent cell-mediated cytotoxicity (ADCC). GMK vaccination induced more consistent high-titer IgM and IgG antibodies than the original GM2-BCG vaccine that had previously improved RFS in stage III melanoma at MSKCC [24]. In this trial, HDI proved to be superior with improved RFS (HR = 1.47, *P* = 0.0015) and OS (HR = 1.52, *P* = 0.009) compared to GMK. Following an interim analysis in April 2000 that showed a mortality benefit for HDI, it was felt to be ethically difficult to continue the GMK intervention, and this trial was closed. Subsequent analysis found that GMK had induced antibody responses in 80% of vaccinated patients, and that those patients who had developed anti-GM2 antibody response showed a trend to improve outcome compared to those without immune response—indicating that a lack of immune response was not to blame. Post hoc intention-to-treat analysis confirmed the improved RFS (HR = 1.49) and OS (HR = 1.38) for HDI.

E2696 was an ECOG-sponsored randomized, phase II trial that enrolled 107 patients with resected stage IIB, stage III, and stage IV disease (including patients with resectable intransit metastases or extracapsular extension of nodal disease [formerly AJCC designated stage IV, M1 disease but currently classified as AJCC stage IIIC disease]) [25]. The trial comprised 3 treatment arms—arm A (GMK plus concurrent HDI), arm B (GMK plus sequential HDI), and arm C (GMK alone). When results were analyzed at a median follow-up period of 24 months, the combination of HDI/GMK appeared to reduce the risk of relapse when compared to GMK alone (HR = 1.75 for C versus A and HR = 1.96 for C versus B). 

In a pooled analysis of all four ECOG-led trials of HDI published in 2004 [26], the survival of patients enrolled in the afore-mentioned E1684, E1690, E1694, and E2696 trials was updated. Survival and relapse-free outcome analysis were calculated based on data from 713 patients randomized to HDI versus observation in E1684 and E1690. This subsequent analysis again showed the benefit of HDI in terms of improved RFS (HR = 1.30, *P* < .006). The significant mortality benefit noted in the first mature report of E1684 was not significant in the pooled analysis (HR = 1.08 for non-IFN-treated versus IFN-treated arms, *P* = 0.42). As mentioned earlier, this observation was qualified by the confounding of E1690 by the crossover of observation-assigned patients (*n* = 37) who developed regional recurrence after assignment to observation and subsequently received HDI and omission of this data from E1694 because the comparator arm was the vaccine GMK rather than observation.

#### 3.3.4. IFN Therapy: Follow-Up Trials Utilizing HDI

Survival analysis in E1684 had noted that the greatest apparent reduction of relapse occurred relatively early, suggesting that the induction phase had a critical role to play. The Hellenic trial [27] attempted to validate this hypothesis prospectively with a phase III study that randomized patients to a modified induction phase of 15 MU/m^2^ HDI only versus the same induction with a modified maintenance phase in which 10 MU (not per m^2^) was administered TIW for maintenance. The noninferiority study design proposed that the one-month treatment would be considered at least as good as the one-year regimen treatment if the relapse rate at 3 years from study entry was no more than 15% higher in the one-month treatment arm. A sample size of 152 patients per treatment arm was planned, and the study enrolled 364 patients in total (182 patients per arm). At a median followup of 5.25 years, there was no statistically significant difference in either median RFS or OS. The trial concluded that at the 5% level of significance the 3-year relapse rate of the one-month group was not 15% higher than the relapse rate of the one-year group. This trial's results have to be interpreted in light of two factors. First, the noninferiority trial design implies that the trial was not powered to detect small differences in RFS between the two arms. Second, the study utilized nonstandard IFN-*α*2b doses of 15 MU/m^2^ for induction and a flat maintenance dose of 10 MU/day rather than the 20 MU/m^2^ induction and 10 MU/m^2^ maintenance doses of the E1684 regimen approved by the FDA.

A more recent US intergroup study (E1697) also attempted to test this hypothesis in patients with resectable intermediate risk melanoma (≥T3 or any thickness with microscopically positive node disease—N1a-N2a). 1150 of a planned 1420 patients were randomized to either 4 weeks of HDI (20 MU/m^2^/day for 5 days weekly) versus observation [28]. This study was closed for futility in 2010 and presented to ASCO in 2011, revealing a lack of any impact upon either RFS or OS with IFN. The study demonstrated a 5-year survival rate for IFN 0.82 (95% CI 0.78–0.86) versus observation 0.85 (95% CI 0.81–0.89) with conditional probability analysis of the study showing a less than 1% chance of showing the desired 7.5% reduction in relapse rate, even if taken to completion. Notably ulceration was present in 36% of patients, and 19% had microscopic node positive disease, so that the risk profile was somewhat less than originally anticipated—leading to concern that the study was underpowered to detect OS/RFS benefit in the segment of patients best suited for it.

Another observation from the pivotal E1684 study was that the greatest improvement in survival (hazard ratio) was seen in patients with clinically negative but pathologically positive nodes (N1 disease). The Sunbelt Melanoma Trial [29] was an ambitious trial designed to evaluate whether patients with a single positive sentinel lymph node biopsy who went on to complete lymph node resection benefited from subsequent HDI. Eligible patients had primary melanomas with Breslow thickness of ≥1.0 mm were subsequently staged with sentinel lymph node (SLN) biopsy. In the intention-to-treat portion (Protocol A) of the study, SLN-positive patients were randomized to either HDI (induction and maintenance per the FDA-approved E1684 protocol) or observation following complete lymph node dissection. This trial never achieved its stated accrual goals and was severely underpowered as analyzed in the interim. The intention-to-treat analysis of Protocol A revealed no significant differences in either DFS (HR = 0.82, 95% CI 0.47–1.40) or OS (HR 1.07, 95% CI 0.65–1.78) between patients randomized to HDI versus observation. (The small numbers here qualify any interpretation of this trial, but the HR of  .82 is identical to the benefit reported for intermediate dosages of PegIFN at the FDA review of that agent, which resulted in its approval based upon early results of that trial this year). This complicated trial also included a secondary protocol (Protocol B) that attempted to assess the utility of molecular staging for SLN specimens. Patients with negative SLN by standard histopathology and immunohistochemistry underwent molecular staging by reverse transcriptase polymerase chain reaction (RT-PCR) to detect melanoma-specific mRNA (tyrosinase, MART 1, MAGE 3, gp100). Patients with SLN-positive disease by RT-PCR were then randomized to observation versus completion lymph node dissection (CLND) versus CLND + INF*α*2b (E1684 induction phase only). Analysis of the Protocol B results found no significant differences in DFS or OS among patients randomized to CLND or CLND + IFN-*α*2b versus observation, but the inadequate numbers accrued to this trial again qualify any conclusions from the study.

The Italian Melanoma Intergroup recently presented data from a randomized phase III study that assessed the utility of a shorter but more intense course of HDI (intensified HDI, IHDI) at ASCO 2011 [30]. 336 patients with stage III disease were randomly assigned to standard HDI therapy or 4 cycles of IFN-*α*2b 20 MU/m^2^ intravenously 5 days a week for 4 weeks every other month (IHDI). At 5 years, the RFS and OS rates in the IHDI arm were 45.8% (95% CI 37.4–53.7) and 60.1% (95%CI 53.0–66.5), whilst the corresponding rates in the standard HDI arm were 44.3% (95% CI 35.7–52.6) and 52.7% (95%CI 44.9–59.8) with no statistically significant difference between the two groups. More importantly, the discontinuation rate and overall toxicity profiles were relatively similar in both groups—suggesting that the shorter but more intensive IHDI regimen may be more feasible than conventional HDI. However, mature survival data has yet to accrue for this combination, and it lacks the validation of conventional HDI.

Considering all available evidence for adjuvant HDI, it is clear that there is a uniform unquestionable improvement in RFS with IFN-*α*, with a smaller but reproducible benefit upon OS evident in two trials of HDI, and meta-analyses of all reported trials of IFN irrespective of dosage. E1684 demonstrated a statistically significant survival benefit that was not reproduced in E1690, where the issue of crossover was recognized and documented; the benefit upon survival was equal to the benefit upon survival in E1694, documented in relation to the vaccine GMK that was at issue until recently. 

The pooled analysis of E1684/E1690/E1694/E2696 and several meta-analyses have found strong evidence for prevention of relapse by IFN, with significant but smaller improvements in overall survival that appears to be greatest in patients with ulcerated primaries and/or patients with node-positive disease—a proposition will be tested in the upcoming EORTC 18081 trial. Taken together, the above results have supported the initial US FDA approval and resulted in sustained approval of high-dose IFN-*α* by the US FDA.

#### 3.3.5. IFN Therapy: Low and Intermediate Dosing and Duration of Therapy

In an effort to improve upon the toxicity of HDI, less intensive regiments were tested by several authors. These included intermediate (5–10 MU/m^2^), low (≤3 MIU/m^2^), and very low (1 MIU/m^2^) dosing regimens, and the trials are summarized in Table 2 (Table 2—phase III studies of IFN-*α* for metastatic melanoma). 

Although some of these trials demonstrated a benefit in RFS for the IFN arm relative to placebo, these differences tended not to be durable. The EORTC (European Organization for Research and Treatment of Cancer) 18952 trial [31] assigned 1388 patients with stage IIB/III disease to one of two intermediate dosing schedules (four weeks of induction with 10 MU five times per week, followed by either 10 MU thrice weekly for one year or 5 MU thrice weekly for two years) versus observation. At the relatively early time point of 4.5 years, patients treated for two years were more likely to be free of distant metastasis than those treated for one year or managed with observation only (47% versus 43% and 40%, resp.). OS was greater in the two-year treatment arm (53%) compared to the one-year arm and observation (48% each). These differences did not reach statistical significance.

Apart from dosage variations, several investigators have experimented with the duration of IFN-*α* therapy, based on observations from small early trials and the large French multicenter trial that suggested that the effect of interferon on RFS disappeared rapidly on cessation of treatment [32]. A meta-analysis of 12 randomized trials by Wheatley et al. [33] had shown that IFN-*α* therapy reduced odds of recurrence and the risk of death compared with observation or vaccination without defining the optimum dose or duration of interferon therapy.

A randomized Phase III Dermatologic Cooperative Oncology Group (DeCOG) study [34] evaluated the utility of the LDI/Dacarbazine (DTIC) in 444 patients with microscopic or macroscopic regional node metastases following surgery and complete lymphadenectomy who were at a high risk of recurrence. Comparator arms were SC 3 MU 3 days a week for 24 months (A) versus SC 3 MU 3 days a week for 24 months + DTIC 850 mg/m^2^ every 4–8 weeks for 24 months (B) versus observation (C). At approximately 4 years of followup, the low-dose IFN combination was associated with an improvement in DFS (HR = 0.69) and OS (HR = 0.62)—the second study to demonstrate a survival benefit for IFN-*α* therapy after E1684. It must be noted, however, that this trial was only powered to assess if DTIC adds any benefit to IFN-*α* and not whether low-dose IFN-*α* therapy was indeed superior to observation. These results are also inconsistent with earlier Austrian, French, and UK studies that have already been cited, which demonstrate no OS benefit of LDI.

A subsequent randomized study by the same group then evaluated LDI therapy in patients with intermediate-high-risk disease (*T* ≥ 1.5 mm) and negative clinical lymph node status [35]. It compared INF-*α* at 3 MU thrice weekly subcutaneously for either 18 months (arm A) or 60 months (arm B). Approximately 75% of all patients had SLN evaluation with a similar rate of positivity in both groups. Relevant prognostic factors including Breslow depth were well balanced between both groups. RFS, DFS, and OS were similar in all 3 groups with no apparent benefit with increasing duration of therapy. 

The recently published Nordic IFN trial was a prospective multicenter randomized phase III trial designed to see if an extended duration of intermediate-dose IFN-*α*2b (IDI) therapy would improve RFS compared to observation [36]. Study investigators compared two different schedules (induction 10 MU SC 5 days weekly for 4 weeks followed by maintenance 10 MU SC thrice weekly for either 12 or 24 months) of IFN-*α*2b to observation for patients with high risk cutaneous melanoma (T4N0M0/TxN1-2M0) with no evidence of distant metastasis or had undergone surgery for regional lymph node metastases. At a median follow-up time of 6 years, the authors found that 1 year of maintenance therapy significantly improved median RFS compared to controls—37.8 months [1-year arm] versus 23.2 months [controls] and 28.6 months [2-year arm] (*P* = 0.0.34). Surprisingly, 2-year therapy did not achieve a significant increase in RFS, where the 2-year therapy in EORTC 18952 had been most effective—although this may be a peculiarity of this trial [30]. Unlike HDI in E1684, the Nordic IFN trial did not record a significant improvement in OS compared with untreated controls.

#### 3.3.6. IFN Therapy: PEG-IFN Therapy and Duration

Pegylated IFN (PEG-IFN) is a form of recombinant human IFN that has been chemically modified by the covalent attachment of a polyethylene glycol moiety that results in sustained absorption and prolonged half-life and has been shown to increase efficacy compared with nonpegylated IFNs in hepatitis C patients [37–39]. 

EORTC 18991 investigated the efficacy and safety of pegylated IFN-*α*2b versus observation in patients with resected AJCC stage III melanoma. Peg-IFN-*α*2b therapy comprised induction dose (Peg-IFN-*α*2b SC 6 *μ*g/kg a week for 8 weeks) followed by maintenance dose (once weekly SC injections at 3 *μ*g/kg for 5 years) [40]. Pegylated IFN-*α*2b (PegIntron, Schering-Plough) was approved by the FDA in October 2009 on the basis of these results. The investigators recently presented 7.6-year follow-up data—which showed an improved RFS in the treatment arm (HR 0.87, 95% CI 0.76–1.00, *P* = 0.05) with no difference in OS/DMFS between treatment and observation arms. Subgroup analysis suggested that patients with microscopic nodal metastasis and ulcerated primaries benefited more from therapy in terms of RFS, OS, and DMFS—an unplanned subset analysis benefit that has been maintained at longer-term followup, where the overall benefit upon relapse-free survival has eroded from 18% to 13% benefit (*P* = 0.01 → 0.05). This observation is slated for testing in EORTC trial 18081. 

Low-dose pegylated IFN-*α*2b was evaluated against LDI in the European Association of Dermato-Oncology (EADO trial) that prospectively enrolled 896 patients with resected stage IIA-IIIB melanoma (*T* ≥ 1.5 mm, without clinically detectable nodal disease) in a phase III trial. Patients were randomized to receive either 36 months of low-dose peg-IFN-*α*2b (100 mcg SC once weekly) or 18 months of LDI (3 MU SC thrice weekly). RFS, OS, and distant metastasis free survival (DMFS) were similar in both groups. Analyses were likely affected by the high dropout rate (72% before study end) secondary to serious adverse events in the peg-IFN arm (44.6% versus 26.6%) [41].

#### 3.3.7. IFN Therapy: Meta-Analyses

Several meta-analyses have attempted to consolidate and review the available outcome data on IFN therapy [9–12].

A 2010 meta-analysis of data from randomized clinical trials (RCTs) published between 1990 and 2008 reviewed a total of 8122 patients, of whom 4362 patients received IFN-*α* [12]. In 12 of the 14 trials, single-agent IFN-*α* was compared with observation, and 17 comparisons (IFN-*α* versus comparator) were generated in total. The meta-analysis showed a statistically significant reduction in recurrence for patients receiving IFN-*α* (HR = 0.82, 95% CI 0.77–0.87, *P* < 0.001). Most interestingly, when analyzed by subgroup, no particular IFN-*α* regimen, IFN-*α* type, TNM disease stage, or study design conferred any statistically significant differences in overall hazard ratio estimates. 

When original data from 12 of the 14 RCTs that assessed the impact of IFN-*α* on OS were used to reassess OS, 4 of the 14 comparators (*n* = 2110) found a statistically significant OS advantage in favor of patients treated with IFN-*α*. Meta-analysis revealed a statistically significant reduction in the risk of death for patients allocated to the IFN-*α* arm (HR for death = 0.89, 95% CI 0.83–0.96, *P* = 0.002). No difference in results was found when original data was substituted for updated data—including the updated OS analysis from E2696 (the original had analyzed DFS). 

The authors concluded that IFN-*α* therapy demonstrated improvement in both RRFS (risk reduction = 18%) and OS (risk reduction = 11%) of patients with high-risk cutaneous melanoma in a statistically significant fashion.

#### 3.3.8. IFN Therapy: Refining the Dose and Duration of IFN in Adjuvant Therapy for Melanoma

While HDI has consistently demonstrated improved RFS, none of the alternative low, very low, or intermediate dosing regimens has demonstrated durable sustained improvements in either RFS or OS. These include the very low dose (1 MU SC every other day) tested in EORTC 18871 (stage IIB/III) [42], low dose (3 MU SC thrice weekly) tested in WHO melanoma program trial 16 (stage III) [43], E1690 (T4, N1) [22], UKCCCR AIM-High trial (stage IIB/III) [44], and the Scottish trial (stage IIB/III) [45]. Whilst an OS and DFS benefit was noted in the LDI arm of the 2008 DeCOG trial [34] that evaluated the combination of low-dose IFN and DTIC in patients with microscopic or macroscopic regional node metastases following surgery and complete lymphadenectomy, it must be noted that this trial was not powered to assess the efficacy of LDI.

When considering the trials that tested the intermediate dose of IFN-*α*, although EORTC 18952 (stage IIB/III) [31] demonstrated a 7.2% increase in DMFS, this was not statistically significant and no sustained OS benefit was observed. 

The Hellenic trial 13A/97 assessed the role of induction phase therapy with modified HDI—several issues including the noninferiority design as well as the use of nonstandard IFN-*α*2b doses in the induction and maintenance phases led several investigators to revisit the issue of an abbreviated HD IFN course [46]. Investigators in Beth Israel Deaconess Medical Center retrospectively identified 86 patients with IIB-IIIA melanoma treated between 2002 and 2009 [47]. Whilst all patients had received standard induction therapy for 4 weeks (IFN-*α*2b IV at 20 MU/m^2^ daily for 5 days per week), maintenance therapy (thrice weekly SC injections at 10 MU/m^2^) duration was either 48 weeks (patients treated prior to January 2006) or 12 weeks (patients treated between January 2006 and January 2008). RFS at 3 years was 80% in the 12-week cohort and 87% in the 48-week cohort (*P* = 0.41), whilst OS at 5 years was 90% in the 12-week cohort and 88% in the 48-week cohort (*P* = 0.99). Study investigators noted that whilst RFS/OS were similar in patients with IIB/IIIA disease, RFS appeared worse in patients with IIC disease. However, despite the statistical nonsignificance of these results, the use of a modified HDI dose-schedule that may increase compliance merits investigation in a prospective randomized trial involving intermediate risk patients and with a control group so that the activity of this regimen can be gauged.

### 3.4. Identifying Prognostic Factors of IFN Therapy for Melanoma

Much of what we have learned in the past two decades suggests that in patients with high-risk disease, adjuvant IFN-*α*2b therapy has a remarkably consistent beneficial effect on RFS but a lesser impact upon survival, especially after 10 years. 

The attenuation in survival benefit over time may be explained by several factors. Firstly, with increasing survival other competing sources of mortality may cause death. Secondly, there is increasing evidence that IFN-*α* plays a role in vascular damage through promotion of antiangiogenesis. High levels of IFN-*α* result in transcriptional repression of IL-1*α* and IL-1*β*, IL-1R1 and VEGF-A thereby altering the balance between endothelial cell apoptosis and vascular repair. [46] Evidence from the rheumatologic literature suggests that this may explain the increased rate of cardiovascular events in patients with active systemic lupus erythematosus [48, 49]. Evidence from multiple intergroup trials buttressed by European data suggests that a certain subgroup of patients (ulcerated node-positive disease) obtain greater benefit with IFN-*α*2b therapy whilst others benefit less, if at all. This suggests that focusing IFN-*α*2b therapy for this group of patients with ulcerated tumors which comprised a subset that was not specified for analysis in prior trials may result in improved outcomes including sustained OS benefits although the fact that prior analysis of multiple ECOG and US Intergroup trials have not identified ulceration as a predictor of improved benefit is of concern. The results of EORTC 18081 are awaited to evaluate this question.

Autoimmune manifestations are a known feature of IFN therapy in chronic viral hepatitis and hematologic malignancies. These include the appearance or increase in titers of autoantibodies or less commonly, the occurrence of overt autoimmune diseases, especially of the thyroid. The prognostic importance of the development of autoimmunity in these diseases is unclear. However, in the melanoma context, the concurrent appearance of autoimmune phenomenon has long been considered a good prognostic factor [50].

In both E2696 and E1694, the development of autoimmunity following IFN-*α* therapy was associated with an improved outcome [51, 52]. However, the first prospectively validated analysis of autoimmunity as a biomarker for IFN response was published by Gogas et al. in 2006 [53]. The Hellenic trial was a noninferiority study designed to assess the importance of the induction phase of HDI therapy. In a substudy of this 364 patient trial, 200 patients had blood samples drawn at baseline, and subsequently at 1, 3, 6, 9, and 12 months of therapy and assayed for various autoantibodies. Interestingly, it was noted that the overall incidence of autoantibodies or autoimmune manifestations amongst patients receiving therapy for one year was greater than amongst those who only had induction therapy—but only barely (28% versus 24%). Strikingly, the patients who developed autoimmune manifestations had better DFS and OS—at a median followup of 46 months, patients with evidence of autoimmunity had improved reductions in the rate of relapse (13% versus 73%) and of overall mortality (4% versus 54 %) compared to those who did not develop autoantibodies. 

Specific human leukocyte antigen (HLA) classes I and II antigens have been associated with greater response to therapy and OS in patients with metastatic melanoma treated with interleukin-2 [54, 55]. Gogas et al. analyzed the Hellenic trial with respect to HLA allele frequencies between patients with and without recurrences after HDI therapy. At a median followup of 70.67 months, the authors noted that HLA-Cw*06-positive patients had a better RFS and OS (*P* = 0.013 and *P* = 0.025, resp.). Even correcting for the presence of autoimmunity, this difference remained statistically significant for improved RFS in the HLA-Cw*06-positive cohort (*P* = 0.020) [56].

Investigators at the University of Pittsburgh and ECOG have evaluated the E2696 and E1694 trials to better understand the prognostic value of autoimmunity induced by HDI. Sera from 103 patients in E2696 and 691 patients in E1694 banked at baseline and up to 3 additional time points were tested by ELISA for the development of 5 autoantibodies. In E2696, autoantibodies were induced in 17 subjects (25%; *n* = 69) receiving HDI and GMK versus 2 (6%; *n* = 34) receiving GMK without HDI (2*P* value =.029). Of 691 patients in E1694, 67 subjects (19.3%; *n* = 347) who received interferon developed autoantibodies versus only 15 (4.4%; *n* = 344) in the vaccine control group (2*P* value < 0.001). In the HDI arms, almost all induced autoantibodies were detected at ≥12 weeks after initiation of therapy. A landmark analysis of E1694-resected stage III patients showed survival advantage associated with HDI-induced autoimmunity that approached statistical significance after adjusting for treatment (HR = 1.54; *P* = 0.072) [52]. Whilst the development of autoimmunity is a useful surrogate to assess response in IFN therapy, an inability to test for it prior to treatment limits its potential in this regard.

Methylthioadenosine phosphorylase (MTAP) catalyses the phosphorylation of methylthioadenosine (MTA), a by-product of polyamine synthesis. Immunohistochemical analysis comparing benign melanocytic nevi to melanomas has shown an inverse association between MTAP protein expression and progression of melanocytic tumors. MTAP also plays a significant role in the activity of signal transducer and activator of transcription 1 (STAT1), an essential component for activation of the interferon *γ* signaling pathway. Utilizing a tissue microarray analysis of 465 unique patients with pigmented lesions (ranging from melanocytic nevi to melanoma metastases), Meyer et al. [57] demonstrated that MTAP expression was significantly associated with OS (*P* < 0.01) and RFS (*P* < 0.05). STAT 1 expression had no significant prognostic relevance in this analysis. Sub-group analysis involving 39 patients whose primary lesions were 1.5–4.0 mm and received adjuvant LDI revealed that patients with MTAP-positive primary melanomas had a significantly longer RFS (*P* < 0.05) and OS (median survival 80 months versus 35 months) compared to patients with MTAP-negative tumors. Despite the small numbers and retrospective nature of the study, this observation bears mentioning given the accumulated data [58–60] surrounding pSTAT3 as a biomarker of melanocytic transformation and the importance of the relative balance of pSTAT1/pSTAT3 in governing melanocyte differentiation.

YKL-40, a mammalian chitinase-like protein, is expressed, and secreted by several types of solid tumors. Retrospective analyses have shown that elevated YKL-40 levels are an independent prognostic factor of RFS and OS in stage I and II melanoma [61, 62] and are correlated with poor survival in patients with metastatic disease. A pooled analysis of 1041 patients from three clinical trials assessing adjuvant IFN in stage IIB-III melanoma (Nordic Study, EORTC 18952, and EORTC 18991) evaluated ELSA-determined YKL-40 levels in serum samples that were collected at study outset, during treatment and at followup every three to six months for up to 10 years. Univariate analysis of baseline YKL-40 levels in 299 untreated patients demonstrated an association of higher levels with short OS (HR = 1.28; 95% CI 1.05–1.57, *P* = 0.015). When serial values were stratified by treatment and analyzed, it was shown that increases in the YKL-40 levels were significantly associated with shorter OS in all treatment arms [63]. 

Other serum biomarkers of interest in melanoma include S100B, melanoma-inhibiting activity (MIA), and tumor-associated antigen 90 immune complex (TA90IC). S100B, an immunohistochemical marker of pigmented skin lesions, has prognostic utility in melanoma—with rising concentrations of serum S100B (above >0.6 *μ*g/L) indicating progression of the disease and a decline indicating response to treatment [52]. S100B levels have been associated with mortality—data derived from E1694 has shown that a high baseline or increasing serum S100B is an independent prognostic marker of risk for mortality in patients with high-risk disease. Swiss and German guidelines recommend determining serum S100B levels in patients with T2 or greater (Breslow >1 mm) lesions every 3–6 months. 

MIA is a growth-inhibiting protein that is strongly expressed in malignant melanomas, but not in benign melanocytic nevi [64]. A German study [65] of 326 patients with melanoma (using a cutoff of 9.8 ng/mL) reported that MIA levels were elevated in 89.5% and 60.0% of patients with stage IV/III disease, respectively, compared to just 5.6% of patients with stage I/II disease. A subsequent study of 373 melanoma patients evaluating the combination of S100B, MIA, LDH, and albumin as biomarkers reported that S100B had the greatest sensitivity for detecting new metastases compared to MIA, LDH, or albumin. TA90IC was compared to MIA and S100B in a prospective 75 patients study involving stage III melanoma patients undergoing adjuvant vaccine immunotherapy following completion of lymph node dissection. Serum samples were drawn before initiation of immunotherapy and at six follow-up time points. Study authors noted that TA90IC was the first marker to become elevated in 29 (57%) followed by MIA, and S100B. Multivariate regression analysis suggested that TA90IC was an independent predictor of survival when elevation occurred between 2 weeks and 3 months, whereas MIA was an independent predictor appearing at 4–6 months. Notably, all the patients in this study had elevated S100B levels (above manufacturer's recommended upper limit of normal) likely secondary to detection of vaccine-related tumor antigen. Whilst S100B, MIA and TA90IC may be useful in assessing prognosis in melanoma, they have not been evaluated as response markers for IFN-adjuvant therapy for melanoma.

### 3.5. Non-IFN-Based-Adjuvant Therapy: Chemotherapy

Non-IFN-based therapies have been investigated in the adjuvant setting in multiple different trials—the most important randomized controlled trials (RCTs) are summarized in (*Tables 3 and 4—phase II/III studies of chemotherapeutic agents in melanoma*).

Chemotherapy whether as a single agent or in combination with other chemotherapeutics, hormonal therapy, or biologic therapy has not shown any improvement in either DFS or OS in any RCT to date except in high-selected patients under special settings (isolated limb perfusion). When biologics were combined with chemotherapy (biochemotherapy) in the metastatic setting, higher response rates and prolongation of median survival were observed although no OS benefit was noted compared to DTIC monotherapy.

The South West Oncology Group (SWOG) has led an intergroup phase III trial of biochemotherapy for 3 months compared with HDI for one year in stage-resectable IIIB and IV patients (S0008). The study arm involves three cycles of cisplatin, vinblastine, DTIC, IL-2, and interferon—with both the IL-2 and interferon being dosed substantively below their individual maximally tolerated doses. This study is therefore better understood as an assessment of the effect of chemotherapy modulated by IFN/IL-2—at present, it remains under analysis. However, the negative result of a recent intergroup study comparing biochemotherapy to polychemotherapy alone has tempered expectations [66].

The apparent benefit of vindesine in treating stage III melanoma in the adjuvant setting was suggested in several single-center studies—but the result has not been reproducible in any RCT. Following suggestion of benefit from small nonrandomized single-institution studies, further studies using megestrol acetate, vitamin A, and nonspecific immunostimulants such as BCG, Corynebacterium parvum, and transfer factor have unfortunately turned in negative results. 

Three trials have assessed the use of adjuvant chemotherapy following surgical resection in high-risk patients—two demonstrated increases in RFS, whilst no benefit was observed when DTIC was combined with BCG in the postoperative setting (E1673) [67]. In the phase III DeCOG trial comparing adjuvant low-dose IFN to LDI/Dacarbazine (DTIC) combination to observation in high-risk patients with regional node metastases following complete lymphadenectomy, a survival benefit was only observed in the low-dose IFN alone arm [34]—however, this trial was not powered to assess the benefit of low-dose IFN over observation.

### 3.6. Non-IFN-Based-Adjuvant Therapy: Vaccine Therapy

Since 1967 when Morton first investigated the use of vaccines to treat patients after surgery, melanoma vaccines have been extensively investigated in the hope of eliciting durable clinical responses with minimal additional toxicity. Vaccines aim to increase immune recognition and enhance antitumor responses through improved antigen presentation resulting in highly durable effector T-cell responses.

Melanoma vaccines can be categorized based on the type of antigen incorporated—peptide, ganglioside, and whole cell/cell lysate. Examples of the former include MART-1/Melan-A, gp100, and tyrosinase—these are melanocyte lineage antigens recognized by cytotoxic T lymphocytes in conjunction with HLA-A2.1 and elicit a direct cytotoxic T-cell response. These T-cell peptide antigens have been studied in large multicenter ECOG trials that have generally recruited pretreated patients with advanced metastatic melanoma. Patients who demonstrated immune responses to any of the peptides develop increased T-cell production of IFN-*γ* and had survival times that were nearly double that of patients who did not develop immunity to 1 or more of the peptide vaccine epitopes. 

MAGE tumor antigens are expressed in a variety of malignancies including melanoma, non-small-cell lung cancer, and head and neck squamous cell carcinoma but are not detectable in normal tissues except for testis and placenta [68]. Whilst recognition of the MAGE-3 antigen is normally limited by HLA haplotype, this can be bypassed by using protein adjuvants that elicit a broader range of T-cell responses. A phase II study utilized this approach to elicit MAGE-3-specific antibody and T-cell responses successfully [69]. A randomized phase III trial involving patients with completely resected stage III melanoma with detectable MAGE-3-specific expression in resected lymph nodes (DERMA study, GlaxoSmithKline) has completed accrual, and results are expected shortly. This trial is unique in having incorporated a tumor tissue profile that appears to predict benefit of vaccine therapy, as preliminarily tested in patients with advanced melanoma who were vaccinated against Mage A3 [70]. The results of this trial are expected in 2014.

Gangliosides are sialic acid-containing glycosphingolipids that are overexpressed on surface of melanocytic cells. It has long been known that de novo responses to the GM2 ganglioside are associated with extended survival in melanoma patients. GM2 with bacillus Calmette-Guerin (bCG) as an adjuvant or combining it with the keyhole limpet hemocyanin (KLH) hapten and a QS21 adjuvant effectively induces antibody responses to GM2 [71, 72]. Both approaches were tested in the phase III setting in E1694 and EORTC 18961 against HDI and observation, respectively. Neither failed to demonstrate any RFS/OS benefit for vaccine therapy—in EORTC 18961, the trial was actually terminated as early evidence suggested that vaccination was ineffective and potentially detrimental. When the final results of this trial were presented in 2010, the authors noted that vaccination resulted in poorer DMFS and OS compared to observation in a nonstatistically significant fashion [73]. 

Administering GM2 with BCG or combining it with the keyhole limpet hemocyanin (KLH) hapten and using a QS21 adjuvant is effective in inducing an antibody response to GM2. However, two large phase III trials (EORTC 18961 and E1694) did not demonstrate a survival benefit when a vaccine using GM2-KLH with the saponin adjuvant QS21 was used in an adjuvant setting [23, 73].

Seven large randomized trials of adjuvant allogeneic melanoma cell-based vaccines have been conducted—none of which have suggested any survival benefit. An Australian study using vaccinia viral lysates in high-risk patients following definitive surgery found that immunotherapy resulted was associated with a statistically nonsignificant increase in RFS (50.9% treated group and 46.8% control group) [74]. A subsequent study evaluated LDI/melanoma lysate vaccine combination (Arm 1) compared against standard HDI (Arm 2) in patients with resected stage III disease [75]. Authors found that the LDI/vaccine combination was associated with similar rates of OS and RFS at 32 months of followup as HDI—61% (LDI/vaccine) versus 57% (HDI) for OS and 50% (LDI/vaccine) versus 48% (HDI) for RFS. 

Morton's studies with a polyvalent vaccine, known commercially as Canvaxin, in stage III melanoma patients was evaluated in a retrospective study [76], and it was suggested that median and five-year OS were higher in vaccinated patients than in nonvaccinated patients. However, when subsequently in a phase III RCT for resected stage III/IV melanoma compared against bCG vaccination, Canvaxin failed to improve either DFS or OS with survival being worse (5% in stage IV and 9% in stage III) likely secondary to vaccine induced clinically significant immunosuppression [77].

### 3.7. Non-IFN-Based-Adjuvant Therapy: Radiation Therapy

The optimal role for radiotherapy (RT) in the treatment of melanoma is highly controversial. Once thought to be a relatively radio-resistant tumor, *in vitro* studies of melanoma cell lines have demonstrated widely differing radiation sensitivities within the same tumor. Available data suggests that melanoma cells behave similarly to late-responding tissues of mesenchymal or ectodermal origin that require greater than standard doses per radiation fraction for most effective cell killing. 

RT can be considered in the treatment of primary disease when local surgical control cannot be obtained for cosmetic or other reasons. If apparently adequate surgical margins are obtained, RT may be used to reduce local recurrence rates if other high-risk features are present. These include melanomas with desmoplastic or neurotropic features and T4 lesions (particularly if ulcerated or associated with satellitosis) as well as head and neck melanomas (especially mucosal melanomas). 

In the initial management of stage III disease, RT is rarely indicated as surgical excision provides superior local control as well as important diagnostic and prognostic information. However, there is abundant evidence to suggest that certain clinicopathologic features strongly increase the risk of locoregional relapse despite adequate surgery by as much as 30–50%. These include extracapsular lymph node extension, involvement of 4 or more nodes, bulky disease (exceeding 3 cm in size), cervical lymph node location, and recurrent disease. However, retrospective phase II data evaluating the use of RT in this instance is inconsistent. A prospective multicenter phase III study, ANZMTG 01.02/TROG 02.01, enrolled 250 patients at high risk of regional recurrence in Australia, New Zealand, and the Netherlands. Following lymphadenectomy, patients were randomized to either observation or regional nodal basin RT (48 Gy in 20 fractions). At a median follow-up time of 27 months, RT use was associated with a statistically significant improvement in locoregional control (HR 1.77, 95% CI 1.02–3.08, *P* = 0.041) [78]. However a survival benefit was not demonstrated, and in fact, survival trends countervailed the relapse-free interval benefit such that it is now uncertain whether there is any role for regional prophylactic RT in operable melanoma, except when surgery has not been possible for clear margins. This study suggests that melanoma is not as radio resistant as once thought and advances several roles for RT in the adjuvant treatment of high-risk stage III disease. Curiously, there was no survival benefit apparent from this intervention and a trend toward adverse survival outcome in the group that received RT compared to observation.

Several questions remain unanswered. Tumors with extracapsular extension (ECE) were excluded from E1684—one of only two trials to have demonstrated a survival benefit for HDI in the adjuvant setting. Whilst several other trials have incorporated patients with ECE and other N3 features, the role of adjuvant HDI is less well defined in this setting. ECOG and the Radiation Therapy Oncology Group (RTOG) planned a randomized trial to compare HDI plus RT (30 Gy in five fractions) to HDI alone for patients with a high risk of locoregional recurrence risk. Unfortunately, this was closed due to lack of accrual. At present, there is no data evaluating the role of HDI in preventing systemic or locoregional recurrence in patients with advanced regional nodal disease, such as ECE, and standard therapy has not yet been defined for these patients.

### 3.8. Non-IFN-Based-Adjuvant Therapy: CTLA4 Blockade

The anticytotoxic T-lymphocyte antigen-4 (anti-CTLA-4) monoclonal antibody ipilimumab (MDX-010; Medarex Inc/Bristol-Myers Squibb) is a fully humanized IgG1 monoclonal antibody that blocks the CTLA-4 receptor that is responsible for transmitting an inhibitory signal to T cells to negatively regulate T-cell activation and proliferation—inhibition results in enhanced T-cell activation and proliferation. 

Tremelmumab was actually evaluated before ipilimumab, and despite encouraging results in phase II studies, it produced negative results in the registration phase III trial against dacarbazine that was closed early. Ipilimumab, however, has been hailed as a game changer in the treatment of melanoma after demonstrating improved survival in a phase III trial of patients with metastatic melanoma that compared ipilimumab alone (at a dose of 3 mg/kg), ipilimumab plus a peptide vaccine against vaccine plus placebo. Compared with the peptide vaccine, ipilimumab showed a near doubling of survival rates at 12 months (46% versus 25%) and 24 months (24% versus 14%) and led to fast-track approval by the FDA [3].

Recently, presented phase III data from a multicenter study of 502 patients with previously untreated metastatic disease that compared ipilimumab (at a dose of 10 mg/kg) with dacarbazine at 850 mg/m^2^ to placebo with dacarbazine demonstrated an overall OS that was durable and sustained at 3 years [4]. Whilst CTLA-4 blockade results in a plethora of immune adverse reactions including potentially fatal colitis, the sustained overall response in 20.8% of patients at 3 years provides an impetus to investigate the use of this agent in the adjuvant setting.

Clinical trials are underway to assess the potential for ipilimumab in the adjuvant setting against HDI in the United States (E1609) and against placebo in Europe (EORTC 18071). The EORTC 18071 trial has completed accrual, and results are expected in 2013-2014.

### 3.9. Future Questions/Conclusion

The recent advances in melanoma immunotherapy and molecular therapy directed against the activating mutation of BRAF have reenergized a field that now has many promising agents for which the benefits in combination with one another are a challenge to assess. Indeed, the prospect of testing combinations in relation to conventional endpoints of OS are daunting, and the adoption of intermediate clinical and laboratory biomarker endpoints is a critical need.

The results of the US intergroup study E1697 suggest that the benefit of IFN therapy requires more lengthy treatment than just the induction phase of the HDI regimen. The question of which subgroups of patients are most likely to derive benefits from IFN therapy is among the most pressing needs, since treatment of only the *∼*30% of patients who derive benefit would treble the therapeutic index of this agent. Previous intergroup studies (E1684, E1690, E1694, EORTC 18952, and EORTC 18991) have suggested that the benefit of IFN is restricted to subpopulations of patients that may be identified by the capacity to develop autoimmunity, or the pathological appearance of the primary (ulcerated primaries and/or microscopic node-positive disease). These bases of focusing therapy are being investigated in current trials such as E1697 and E1609 in which the immune responses of patients are being evaluated, as well as the prospective EORTC trial 18081 that will test the benefit of 2 years of pegylated IFN compared to observation in patients with ulcerated stage II primary melanomas ≥1 mm. The identification of the biomarkers of subpopulations of patients that are more responsive will provide further insights into the mechanisms of interferon antitumor activity.

The exciting long-term survival benefits seen in patients with metastatic melanoma treated with CTLA-blockade therapy have raised expectations that this therapy may have even greater benefits in the adjuvant setting. Multiple intergroup and other trials are investigating the role of ipilimumab in this setting including ECOG 1609 (ipilimumab versus HDI after complete resection of high-risk Stage III/IV melanoma), and EORTC 18071 (ipilimumab versus placebo after complete resection of high-risk stage III melanoma) and these results are eagerly awaited. 

Given the low rate of response and high cost of treatment with biologics including ipilimumab and IFN, prognostic biomarkers that may predict therapeutic response to immunotherapies remain an area of active ongoing investigation. Unpublished data from the MDX010–20 study revealed that absolute lymphocyte counts (ALC) drawn whilst on treatment appear to increase in a dose-dependent fashion with ipilimumab therapy—an effect that was observed in both the ipilimumab/vaccine and ipilimumab monotherapy arms. High-baseline ALCs were associated with an improved outcome, and changes in the ALC appeared to correlate with overall survival benefit. Such retrospective analyses, however, need to be interpreted cautiously; studies without a negative control arm can at best estimate the prognostic utility of a biomarker, for example, predict OS. Optimal biomarkers that predict treatment effect need to be prospectively evaluated in studies with control arms. In metastatic disease, Hamid et al. [79] have reported the improved outcome of ipilimumab therapy among patients with elevated tumor infiltrating lymphocyte counts (TIL) and elevated Treg and IDO levels at pretreatment biopsy. The neoadjuvant setting, where there is access to tumor tissue both before and after therapy, provides an ideal opportunity to identify immunologic and histologic correlates of tumor response. Data from an existing neoadjuvant study in which patients received ipilimumab preoperatively followed by lymphadenectomy, and 2 additional doses of maintenance ipilimumab showed a significant increase in the frequency of circulating CD4 +CD25hi + Foxp3 + regulatory T cells [80], a finding independently confirmed by investigators from the Moffit Cancer Center using samples derived from patients treated with ipilimumab on an adjuvant trial [81]. Further analysis comparing baseline and 6-week tumor samples is ongoing.

With exciting results demonstrated in the registration study against dacarbazine in metastatic melanoma, the use of oncogenic BRAF inhibitors such as vemurafenib in the adjuvant setting has been raised. However, the significant progression-free and overall survival benefit observed was tempered by the realization that resistance is rapidly acquired within several months of treatment. Concurrent inhibition of downstream targets of the MAP kinase signaling pathway such as MEK could be a potential solution to this problem.

The year 2011 will likely be remembered as one in which treatments for melanoma dominated news broadcasts all over the world. The optimal combination and sequence of these active agents to derive lasting disease and progression-free survival and maybe even elicit a cure will be the subject of intense investigation in the upcoming years. This will likely be achieved by rationally combining immunotherapy with other forms of targeted and cytotoxic chemotherapies. It is hoped that the impetus provided by recent advances will translate into objective benefits for our patients.

## Figures and Tables

**Table 1:** Phase II trials of IFN-*α* for metastatic melanoma.

Study reference	No. of enrolled patients (followup)	Therapy and IFN subspecies	Dose—treatment arm (MU/m^2^)	Schedule—treatment arm	ORR	CR	PR
Ernstoff et al. 1983 [82]	17	*α*2b	10–100	5 d/week × 1 month	N/A	N/A	2

Creagan et al. 1984 [83]	23	*α*2a	50	Thrice weekly × 12 weeks	20	1	5

Creagan et al. 1985 [84]	350	*α*2a + cimetidine	50	Thrice weekly × 12 weeks	23	0	8

Creagan et al. 1984 [85]	31	*α*2a	12	Thrice weekly × 12 weeks	23	3	4

Legha et al. 1987 [86]	62	*α*2a	1st arm—escalating (3–36 × 10^6^ U/d) 2nd arm—fixed dose (18 × 10^6^ U/d)	1st arm—daily during induction followed by thrice weekly 2nd arm—thrice weekly	1st—12.9% 2nd—16.1%	1st 0 2nd 0	1st—9.7% 2nd—6.5%

Hersey et al. 1985 [87]	200	*α*2a	15–50	Thrice weekly	10	2	0

Neefe et al. 1990 [88]	97	*α*2a	Escalating: 3 to 36 × 10^6^ U	Daily for 10 days then 70 days total	8	6	2

Dorval et al. 1986 [89]	22	*α*2b	10	Thrice weekly	24	2	4

Coates et al. 1986 [90]	15	*α*2a	20	5 d/week every 2 weeks	0	0	0

**Table 2:** Phase III studies of IFN-*α* for metastatic melanoma.

Study reference	No of patients eligible for analysis	TNM stage	Therapy and IFN subspecies	Dose and schedule—treatment arm	Median followup at time of reporting (yrs)	Median DFS (mths)	Median OS (mths)	% Node- positive
				High dose				

NCCTG 83-7052 [20]	262	II–III (T2-4N0M0/TanyN + M0)	IFN-*α*2a versus observation	IM 20 MU/m^2^ thrice weekly for 4 months	6.1	NS	NS	61

ECOG E1684 [21]	287	II–III (T4N0M0/TanyN + M0)	IFN-*α*2b versus observation	IV 20 MU/m^2^ 5 days a week for 4 weeks → then →SC 10 MU/m^2^ 3 days a week for 48 weeks	12.6	6.9	S (at 6.9 yrs) NS (at 12 yrs)	89

ECOG E1690 [22]	642	II–III (T4N0M0/TanyN + M0)	IFN-*α*2b—high dose versus low dose versus observation	High dose: IV 20 MU/m^2^ 5 days a week for 4 weeks → then → SC 10 MU/m^2^ 3 days a week for 48 weeks Low dose: SC 3MU/m^2^ 2 days a week for 2 years	6.6	4.3	NS	74

ECOG E1694 [23]	774	II–III (T4N0M0/TanyN + M0)	IFN-*α*2b versus GMK vaccine	IV 20 MU/m^2^ 5 days a week for 4 weeks → then → SC 10 MU/m^2^ 2 days a week for 48 weeks	2.1	62% (2yr) versus 49%	78% versus 73%	77

ECOG E2696 [25]	107	II–III–IV (stage IV: resectable meta static disease)	IFN-*α*2b with GMK vaccine with and without induction	Induction: IV 20 MU/m^2^ 5 days a week for 4 weeks → then →SC 10 MU/m^2^ 3 days a week for 48 weeks No induction: SC 10 MU/m^2^ 3 days a week for 48 weeks	2.4	S	S	Not available

				Intermediate dose				

EORTC 18952 [31]	1388	II–III (T4N0M0/TanyN + M0)	IFN-*α*2b for 1 yr versus 2 yrs versus observation	IV 10 MU 5 days a week for 4 weeks → then →SC 10 MU 3 days a week for 1 year *OR* SC 5 MU 3 days a week for 2 years	1.6	7.2% (NS)	5.4% (NS)	74

EORTC 18991 [91]	1256	III (TanyN + M0)	PEG IFN-*α*2b versus observation	SC 6 *μ*g/kg/week for 8 weeks → then →SC 3 *μ*g/kg/week for 5 years	3.8	45.6% versus 38.9% (NS)	NS	100

				Low dose				

Austrian melanoma cooperative group (AMCG) [92]	311	II (T2-4N0M0)	IFN-*α*2a versus observation	SC 3 MU 7 days a week for 3 weeks → then →SC 3 MU 3 days a week for 1 year	3.4	S	Not available	0

French melanoma cooperative group (FCGM) [93]	499	II (T2-4N0M0)	IFN-*α*2a versus observation	SC 3 MU 3 days a week for 18 months	>3	0.74 (HR), S	0.70 (HR), S	0

WHO melanoma program trial 16 [94]	444	III (TanyN + M0)	IFN-*α*2a versus observation	SC 3 MU 3 days a week for 36 months	7.3	NS	NS	100

Scottish melanoma cooperative group [45]	96	II–III (T3-4N0M0/TanyN + M0)	IFN-*α*2a versus observation	SC 3 MU 3 days a week for 6 months	>6	NS	NS	Not available

EORTC 18871/DKG 80-1 [42]	728	II–III (T3-4N0M0/TanyN + M0)	IFN-*α*2b versus IFN-*γ* versus ISCADOR M versus observation	IFN-*α*2b: SC 1 MU every other day for 12 months IFN-*γ*: SC 0.2 mg every other day for 12 months	8.2	NS	NS	58

UKCCCR/AIM HIGH [44]	674	II–III (T3-4N0M0/TanyN + M0)	IFN-*α*2a versus observation	SC 3 MU 3 days a week for 24 months	3.1	NS	NS	Not available

DeCOG [95]	840	III (T3anyN + M0)	IFN-*α*2a	SC 3 MU 3 days a week for 18 months (A) versus 5 yrs (B)	4.3	81.9% versus 79.7% NS	85.9% versus 84.9% NS	Not available

DeCOG [34]	444	III (TanyN + M0)	IFN-*α*2a	SC 3 MU 3 days a week for 24 months (A) versus SC 3 MU 3 days a week for 24 months + DTIC 850 mg/m^2^ every 4–8 weeks for 24 months (B) vesus observation (C)	3.9	HR: 0.69 (A) versus 1.01 (B) versus 1.0 (C)	HR: 0.62 (A) versus 0.96 (HR) (B) versus 1.0 (C)	100%

Keys: NS—not significant; S—significant; HR—hazard ratio.

**Table 3:** Phase II/III studies of chemotherapeutic agents in melanoma.

Study reference	No. of patients eligible for analysis (followup)	TNM stage	Treatment arm	Median followup at time of reporting (yrs)	OS
Veronesi et al. 1982 [96]	931	II/III	DTIC BCG DTIC + BCG Obs	5	NS

Lejeune et al. 1988 [97]	325	I, IIA, IIB	DTIC Levamisole placebo	4	NS

Fisher et al. 1981 [98]	181	II/III	CCNU Obs	3	NS

Koops et al. 1998 [99]	632	II/III	Isolated limb perfusion + hyperthermia Obs	6.4	BS

Keys: NS—not significant; S—significant; HR—hazard ratio.

**Table 4:** Phase II/III studies of newer targeted agents.

Study reference	No. of patients eligible for analysis (followup)	Study design	Primary endpoint	Dose and schedule—treatment arm	ORR/OS	PFS (mths)	HR (95% CI)
BMS 008 [100]	155	Phase II, open-label, single arm	Dose finding	Ipilimumab—10 mg/kg	47% (1 yr)	N/A	N/A

BMS 022 [101]	217	Phase II, randomized, double blind	To evaluate the efficacy of three dose levels of ipilimumab	Ipilimumab—10 mg/kg	48% (1 yr)	N/A	N/A

BMS 007 [102]	115	Phase II, randomized, double blind	To evaluate the rate of grade 2 + diarrhea	Ipilimumab—10 mg/kg	51% (1 yr)	N/A	N/A

Medarex MDX010-20 [3]	676	Phase III, randomized, double blind	ORR, subsequently amended to OS	Ipilimumab—3 mg/kg	Ipi alone: 10.1 mths (95% CI 8.0 to 13.8) Ipi + GP-100: 10.0 mths (95% CI 8.5 to 11.5) GP-100 alone: 6.4 mths (95% CI 5.5 to 8.7)	Ipi alone: 2.86 mths (95% CI 2.76 to 3.02) Ipi + GP-100: 2.76 mths (95% CI 2.73 to 2.79) GP-100 alone: 2.76 mths (95% CI 2.73 to 2.83)	Ipi alone (compared to GP-100 alone):0.66 (95% CI 0.51-0.87) Ipi + GP-100 (compared to GP-100 alone):0.68 (95% CI 0.55-0.85)

BMS 024 [103]	502	Phase III, randomized, double blind	OS	Ipilimumab + DTIC: Induction—IPI 10 mg/kg + DTIC (850 mg/m^2^) q3 weeks for 4 doses Maintenance—IPI 10 mg/kg + DTIC (850 mg/m^2^) q12 weeks	Ipi + DTIC: 47.3% (1 yr), 28.5% (2 yr), 20.8% (3 yr) DTIC alone: 36.3% (1 yr), 17.9% (2 yr), 12.2% (3 yr)	Ipi + DTIC: 2.8 DTIC alone: 2.6	Ipi + DTIC: OS 0.72 PFS 0.76

BRIM 2 [104]	132	Phase II, open label	BORR	Vemurafenib (PLX-4032) 960 mg twice daily orally	BORR: 52.3% CR: 2.3% PR: 50%	6.2	N/A

BRIM 3 [5]	675	Phase III, randomized, double blind	OS	Vemurafenib (PLX-4032) 960 mg twice daily orally	PLX-4032: 84% (6 mos) DTIC alone: 64% (6 mos)	PLX-4032: 5.3 DTIC alone: 1.6	Death 0.37 (95% CI 0.26 to 0.55) Progression 0.26 (95% CI 0.20 to 0.33)

Key: N/A—not applicable.
